# Challenges experienced by postgraduate nursing students at a South African university

**DOI:** 10.4102/hsag.v23i0.1107

**Published:** 2018-03-29

**Authors:** Yolanda Havenga, Malmsey L. Sengane

**Affiliations:** 1 Department of Nursing Science, Sefako Makgatho Health Sciences University, South Africa; 2 Adelaide Tambo School of Nursing Science, Tshwane University of Technology, South Africa

## Abstract

**Background:**

The increase in nurses enrolling in postgraduate programmes as well as the need to improve their completion requires academics to establish environments conducive for postgraduate studies. The challenges experienced during postgraduate studies have to be identified to establish conducive environments.

**Objective:**

The objective of this study was to explore and describe the challenges experienced by postgraduate nursing students enrolled in postgraduate coursework and research programmes at a South African university.

**Methods:**

An exploratory, descriptive and qualitative design was used. The study was contextual in nature. Purposive sampling was used. Fifteen honours, master’s and doctoral students participated in the study. Data were analysed through qualitative content analysis and measures to ensure trustworthiness, and ethical implementation of the study were implemented.

**Results:**

Three themes with categories were identified, namely personal challenges (i.e. finances, employment, family and accommodation), academic and institutional challenges (i.e. workload and time constraints, contact sessions, subject information and assessment) and research-related challenges (i.e. information literacy, supervisory relationship and supervisory structure and process).

**Conclusion:**

Institutional support addressing personal, academic and research-related challenges should be provided to enhance student experiences and completion.

## Introduction

Internationally, there has been a gradual increase in the number students enrolling into postgraduate programmes (Symons 2001). This has also been the case for postgraduate nursing programmes (Honey, North & Gunn 2006). The increased interest by nurses to enrol for postgraduate studies is motivated by internal and external motivators (Cleary, Hunt & Jackson 2011; Honey et al. 2006; Perna 2004).

On a professional level, nurses are motivated to obtain higher qualifications as it enables them to progress to more senior positions, enhances their occupational status, improves working conditions, decreases the likelihood of unemployment and enables salary increases and higher lifetime earnings (Cleary et al. 2011; Honey et al. 2006; Perna 2004). Personal motivation is related to enjoyment of the learning experience and increased social status (Perna 2004). This increased motivation for postgraduate qualifications in nursing creates a demand for it to be offered (Honey et al. 2006) and increased capacity to do so at universities.

In South Africa, the introduction of the Occupation Specific Dispensation (OSD) in 2007 (Public Health and Social Development Sectorial Bargaining Council 2007) for public sector employees was first rolled out with nurses. Since the implementation of the OSD, increased interest has been observed by the authors among nurses obtaining those specialised nursing qualifications that would enable them to access the OSD.

This increased motivation for postgraduate degrees and post-basic diplomas create a demand for it to be offered at universities (Honey et al. 2006). Postgraduate programmes are considered conduits through which universities develop research capacity and also generate advanced skills needed (Mutula 2011). There is a need for nurses prepared in specialist and advanced nursing knowledge and skills in various nursing specialities (Honey et al. 2006). By enhancing the competency of nurses through postgraduate programmes, nursing would be enabled to meet the complex and global health needs of individuals, families and communities (Mutula 2011), enhance the quality of nursing care and address the shortage of health professionals (Essa 2011).

In South Africa, nursing as a practice profession requires both practice experts and nursing scientists to expand the scientific basis of patient care. Doctoral and master’s education programmes prepare nurses for advanced practice, leadership and scientific enquiry (American Association of Colleges of Nursing 2006). Furthermore, it is essential that new nurses are prepared with higher academic qualifications such as doctoral degrees in order to replace the leaders who will retire (Cleary et al. 2011). Unfortunately, master’s and doctoral degree programmes in nursing do not produce a large enough pool of postgraduate degree nurses to meet the demand (Rosseter 2012).

The pool could be increased in part by improving the enrolment, retention and completion of postgraduate students. Student retention and completion are key concerns at institutions of Higher Education both in South Africa and internationally (Essa 2011; Maasdorp & Holtzhausen 2011). Higher education institutions (HEIs) are under pressure to ensure acceptable completion rates and research outputs against which their success and efficacy are measured (Essa 2011) and subsidised (Department of Higher Education and Training 2015). In the National Plan for Higher Education, planning was done for the increase of graduate enrolments and outputs at the master’s and doctoral levels (Department of Education 2001). Higher education institutions were required to indicate their strategies to improve their graduate outputs (Maasdorp & Holtzhausen 2011).

## Problem statement

Challenges and negative experiences influence postgraduate students’ non-completion (Choo 2007; Essa 2011). Academics are responsible for establishing and maintaining academic environments that are conducive to learning and research by postgraduate students (Maasdorp & Holtzhausen 2011), and to ensure that postgraduate students complete their studies within a reasonable time (Haksever & Manisali 2000). It is therefore imperative that HEIs and nursing departments explore and address the context-specific challenges related to the retention and completion of postgraduate nursing students. The authors heard of challenges postgraduate students were experienced, and were concerned about the dropout and slow completion by these students. The specific university management indicated that it was necessary to attract a pool of young researchers and provide research-conducive environments for postgraduate students where they would be able to flourish. Postgraduate training and support were proposed to assist with creating more conduce environments (Anon 2011). In order to provide such support and create environments where postgraduate students could flourish, needs and challenges have to be understood (Benshoff, Cashwell & Rowell 2015). The authors set out to understand the context-specific challenges experienced by these postgraduate nursing students in a specific university, as these challenges were not yet explored and seemed to affect these postgraduate students.

## Definition of terms

### Challenges

A challenge can be referred to as a ‘difficult task’. It is something that requires great physical and mental effort to be done successfully and thus tests a person’s ability (Cambridge University Press 2017a). In this study, the term ‘challenge’ refers to that which tests postgraduate students’ abilities to succeed in their nursing studies - a situation they are faced with that needs great mental or physical effort in order to be done successfully and therefore tests the post graduate student’s ability.

### Postgraduate nursing students

A postgraduate refers to a person who already has one degree and studies at a university for a more advanced degree (Cambridge University Press 2017b). In this study, a postgraduate nursing student refers to a student who studies for a more advanced nursing qualification such as honours, master’s and doctoral degrees on National Qualification Framework (NQF), levels 8, 9 and 10 (Council on Higher Education 2013).

### University

A university is the place where a person studies for an undergraduate (first) or a postgraduate (higher level) degree (Cambridge University Press 2017c). In this study, a university refers to a specific HEI where nursing students study towards higher level degrees.

## Methods and material

### Research design

An exploratory, descriptive and qualitative design was used (Polit & Beck 2012), as the researchers attempted to gain a first-hand and holistic understanding of the challenges experienced by the participants in relation to their postgraduate studies. The study was contextual in nature. The qualitative descriptive design is based on the constructivist paradigm and is eclectic, as it uses or adapts research methods from other qualitative research designs (Polit & Beck 2012).

### Setting

The study was contextual in nature (Botma et al. 2010) and was conducted in a nursing department of a specific university situated in South Africa. The nursing department offers various residential postgraduate programmes on a part-time and full-time basis. The postgraduate degree programmes offered are honours (NQF 8), master’s (NQF 9) and doctoral degrees (NQF 10) (Council on Higher Education 2013) in the following specialisations: Midwifery, Psychiatric Nursing, Community Health Nursing, Nursing Management and Nursing Education. Coursework implied attendance of classes for a 2-week period four times per year, writing tests and examination papers, and practical nursing skills being assessed. All postgraduate students complete a research component and are allocated a supervisor and a co-supervisor to guide them through the research process.

### Population and sampling

The study population consisted of all postgraduate students registered during 2011 for a nursing degree at the university. There were 56 postgraduate students registered in 2011, consisting of 15 honours degree students, 27 master’s degree students and 14 doctoral degree students. Purposive sampling was used (De Vos et al. 2011; Polit & Beck 2012) by sending the request to write a narrative report via email to all registered postgraduate students who had been registered for a minimum of 6 months and had an email address. All 56 students had been registered for a minimum of 6 months, as registration took place during January 2011 and data were collected in October–November 2011. A minimum of 6 months registration as a postgraduate student would ensure that all participants had drafted a proposal and had contact with their supervisors. They also would have attended a minimum of two of the four contact sessions for the year. Sampling continued and data were collected until saturation was achieved (Polit & Beck 2012), evidenced by repetition of themes.

The total number of participants was 15. The number of students per programme and whether they were full-time or of part-time are presented in Table 1.

**TABLE 1:** Demographic characteristics of participants.

Programme	Number of students	Full-time/part-time
Doctoral	3	3 Part-time
Master’s	7	5 Part-time 2 Full-time
Honours	5	3 Part-time 2 Full-time

### Data collection

Participants were requested via email to write a self-reporting narrative on the central question: Please describe the challenges you experience as a postgraduate student. In order to increase credibility of data and to determine the appropriateness and usefulness of the central question, it was piloted with two participants who formed part of the population, but were not included in the main study (De Vos et al. 2011). After piloting, the question was deemed appropriate to collect the data that would answer the research question and was used to gather the data.

Each student was sent a participant information leaflet, a consent letter, biographical information questions and the central question, and was asked to write the self-reporting narrative. The initial email was followed up by three reminder emails. Raw data were received by a research assistant via email.

### Data analysis

The research assistant combined all data and grouped them under the headings ‘honours students’, ‘master’s students’ and ‘doctoral students’, after which the data were provided to the two researchers who conducted the analysis. The data were analysed by using qualitative content analysis - a method ascribed to more general qualitative methods (Polit & Beck 2012).

All participant responses were read as a whole. Then the student challenges were read and analysed separately for the honours, master’s and doctoral students. The themes and categories of the three programmes were compared and similarities and unique experiences were identified. The findings for all the participants were then combined. The frequency whereby participants mentioned challenges during the interviews was counted to highlight the categories that were the greatest and the least challenging for students of different programmes and for students who were part-time and full-time (Table 2). The data were analysed by both authors and consensus was reached on the identified themes and categories.

**TABLE 2:** Frequency of challenges mentioned in the data.

Themes and categories	Honours		Masters		Doctoral		Part-time		Full-time		Total
	AF	RF (%)	AF	RF (%)	AB	RF (%)	AF	RF (%)	AF	RF (%)	AF
**Personal challenges**	4	22	12	67	2	11	11	61	7	39	18
Finance	0	0	4	80	1	20	2	40	3	60	5
Employment	3	38	4	50	1	12	8	100	0	0	8
Family	0	0	3	100	0	0	1	34	2	66	3
Accommodation	1	50	1	50	0	0	0	0	2	100	2
**Academic and institutional**	12	57	9	43	0	0	10	48	11	52	21
Workload and time constraints	2	40	3	60	0	0	2	40	3	60	5
Contact sessions	2	40	3	60	0	0	3	60	2	40	5
Subject information and assessment	8	73	3	27	0	0	5	45	6	55	11
**Research**	1	11	1	11	7	78	8	89	1	11	9
Information literacy	1	100	0	0	0	0	0	0	1	100	1
Supervisory relationships	0	0	0	0	3	100	3	100	0	0	3
Supervisory structure and process	0	0	1	20	4	80	5	100	0	0	5
**Total**	**17**	**35**	**22**	**46**	**9**	**19**	**29**	**60**	**19**	**40**	**48**

Notes: Actual frequency (AF) represent the number of times the theme (in bold) was mentioned in the interviews. Relative frequency (RF) represents the percentage of the total actual frequency a theme (in bold) and a category was mentioned. Lines in bold represent the total AF and RF for each theme, respectively, and in the last row, for all the themes combined.

AF, actual frequency; RF, relative frequency.

### Trustworthiness

Quality of the study was enhanced by implementing specific strategies based on Lincoln and Guba’s criteria (1985). To enhance trustworthiness of the study, *credibility* of the study was enhanced by purposive sampling, ensuring saturation of data, and analysis triangulating by two researchers analysing the data and reaching consensus on the identified categories (Holloway & Wheeler 2010:308). *Dependability* and *confirmability* of data were enhanced through well-kept documentation and transparency in the methodology, data analysis and conclusions. *Transferability* was enhanced by obtaining responses from 15 participants and confirming that saturation was achieved. Written responses by interviewees and including verbatim quotes from participants in the description of findings enhanced the *authenticity* of the study (Holloway & Wheeler 2010; Polit & Beck 2012).

### Ethical consideration

The Belmont report’s ethical principles and guidelines for the protection of human subjects of research were used to guide the study. These principles included respect for persons, beneficence, non-maleficence and justice (Polit & Beck 2012). Ethical clearance was obtained from the Research Ethics Committee of the specific university and permission was obtained from the head of the Nursing Department. A research assistant engaged with participants via email so that their responses could not be linked to specific names. The research assistant was a postgraduate student who did not participate in the study and agreed to keep the information confidential. All students were informed that participation was voluntary and that they could withdraw from the study without any consequences to them. Anonymity and confidentiality were ensured by omitting names from any documents. The participants were also informed that the results of the study may be published.

## Findings

Three main themes were identified in the data, namely personal, academic and institutional, and research-related challenges. There were 48 mentions of challenges by the 15 participants. The most frequently mentioned challenge was academic and institutional challenges (21 times), followed by personal challenges (18 times) and research-related challenges (9 times).

For each of the three themes, categories were identified as indicated in Table 2.

Table 2 furthermore indicates the actual (AF) and relative frequency (RF) with which challenges were mentioned for each theme and category. Doctoral students focussed more on supervisory challenges, master’s students more on personal challenges, and honours students more on academic and institutional challenges. It must however be considered that the number of participants per category were not equal.

Each of the themes will now be discussed.

### Theme 1: Personal challenges

Postgraduate students reported that they experienced various personal challenges during their enrolment for postgraduate studies which were related to finances, employment, family and accommodation. Personal challenges are challenges that pertain to the student in his or her personal environment. Students in all three programmes mentioned personal challenges.

#### Finances

Financial challenges related to travelling expenses, student fees, printing, copying, data bundle purchases and money to conduct the research such as travelling to data collection sites and classes.

Participants also experienced challenges in paying student fees, which they perceived as exorbitant, as a master’s student explained: ‘The fees are very expensive for no clear reason’ (part-time, master’s student). The participants reported difficulty in accessing scholarships, as they had no knowledge about funding opportunities and were not certain how to access this information.

A further financial challenge was reported on printing of assignments and journal articles which was a hugely costly exercise as explained by a full-time master’s student. The part time master’s and honours students also reported that accessing electronic material and their emails as students living off campus, was costly, as they had to purchase data bundles to access the information.

A part time doctoral student explained the cost of travelling for supervision: ‘The cost of travelling more than 400 km for a [supervision] contact session of less than an hour …’ With regard to the fees, one part time master’s student said: ‘The fees are very expensive for no clear reason.’

The financial burden when living away from home and studying was explained as follows: ‘As a full-time student, I meet financial constraints to cover printing costs, photocopying costs, meals and all other costs as everything requires money’ (full-time, master’s student 3). Another full-time master’s student explained: ‘The study is very costly, e.g. using internet for consultation, and driving around looking for patients as [anonymous hospital] does not have patients anymore.’ This participant is referring to the patient interventions that have to be completed for the clinical practical part of both the honours and master’s programmes.

#### Employment

The only category mentioned by all three programmes was employment challenges (see Table 2). Employment challenges were related to the high workload at their places of employment and some students not being granted leave or study leave to pursue their postgraduate studies. These students often did so by using their personal leave or working night duty prior to attendance so that they could use their days off. A part-time master’s degree student explained: ‘Part time makes it hard for me to manage my schooling and work.’ Workload and workplace arrangements were not mentioned as a challenge for full-time students (see Table 2), as they were granted study leave from their places of employment.

The challenge with regard to balancing academic and employment responsibilities was explained by one of the participants in the following quotations: ‘Huge workload at the place of employment, which does not allow for such a pattern of contact supervision and other massive academic and administrative programmes’ (part-time, doctoral student). Another doctoral student said:

I raised my concern about the workload at my place of employment (with supervisor) and we both felt that sabbatical leave could be an answer although it’s beyond our control. (Part-time, doctoral student)

#### Family

The participants felt that they had to attend to their family responsibilities such as their children’s needs which impacted their time, energy and ability to focus on their studies. Some participants experienced feelings of guilt as they were not always available when their children required their assistance.

As explained by participants in the following quotations: ‘When at home I still have to study and the children feel left out’ (full-time, master’s student). Two students said: ‘It comes with a price of not having sufficient time for your family’ (part-time, master’s student) and ‘I’m too tired when I get home, I don’t have enough time to do school work with the kids’ (part-time, master’s student).

#### Accommodation

One of the challenges experienced by part-time and full-time students was the lack of available accommodation on campus for students. Many students were not living in the same province where the university was situated and had to travel and sleep over to attend contact sessions. They had to rely on family members or other students, or make use of guest houses in the area for accommodation. As explained by one of the participants:

I have a challenge of finding accommodation inside the campus. I have to look around the nearby location for accommodation. The standard of living conditions is too low outside the campus. (Full-time, honours student)

### Theme 2: Academic and institutional challenges

The academic and institutional challenges were related to the academic workload and time constraints, the structure of contact sessions, subject information and assessment process. These challenges were mentioned most frequently by honours students followed by master’s students. The mention of academic and institutional challenges was near similar between part-time and full-time students.

#### Workload and time constraints

The participants reported on the high workload because of the large amount of theoretical and practical requirements they had to adhere with limited time to do so. They also expressed frustration about the limited time to interact with fellow students. The high workload, programme information and assessment matters were only mentioned by the honours and master’s students, as doctoral students did only research and matters such as practical objectives, study guides and assessments were not relevant to them (see Table 2).

One participant explained:

The challenge that I have experienced is that this course that I’m doing needs more time and that to be a part time student makes it hard for me to manage my schooling and work. (Part-time, master’s student)

Another participant said: ‘Time for practica for part timers is very limited and it is difficult to complete all the practical work in time’ (part-time, master’s student). Students suggested that for the required practical competencies to be more manageable, the requirements had to be reduced: ‘The practical should be reduced by at least half’. (Part-time, honours student)

Students expressed their frustration about the lack of student interaction and support between peers in class which they attributed to time constraints as explained by the following master’s and honours degree students:

[There is] no time to come together with colleagues to discuss the coursework. (Part-time, honours student)

Limited time of interaction with fellow scholars due to limited time at campus together. (Part-time, masters student)

#### Contact sessions

The rigidity in the method of offering contact sessions challenged students. Some of the honours and master’s students were permanently employed and could not always avail themselves on the set dates for contact on campus because of their work commitments.

There was a need for restructuring the offering of the masters and honours programmes with inclusion of after-hour classes as seen in the following quotes:

‘Classes should be offered after-hours, e.g. 17h00–20h00 in the evenings or even on Saturdays’. (Full-time, honours student) [The programme] ‘lacks after-hours/Saturday classes’. (Part-time, master’s student).

Some of the students expressed their challenges regarding the lack of contact with their subject lecturers during clinical practical placement and experienced contact once a week during these times as inadequate. They would prefer a clinical mentor to be available throughout during their practical placement as these master’s and honours students explained:

Another challenge is [*the*] lack [*of*] physical contact with [*a*] clinical mentor at [*the*] clinical area, there is no physical contact when stuck; the only way to communicate is through technology [*email*]. (Full-time, honours student)

#### Subject information and assessment

Challenges related to the adequacy of the information students received about the programme were experienced. Some students indicated to have received inadequate programme information in some of the programmes which impacted their ability to prepare and plan to address the high workload.

As explained:

‘The study guides are not adequately prepared and organised’. (Part-time, master’s student)

The same student said:

‘Some lecturer[s] does [do] not have study guides which describe their year plan such as assignments and their submission dates’.

Some students were challenged by the delayed feedback they received from assessors or supervisors as well as by the structure of feedback given. They explained that assessment and research feedback did not promote their growth and did not give them an opportunity to improve by the next assessment. One student said: ‘The feedback being given [in some] module[s] does not develop one professionally as you are not told what you were supposed to do to improve’ (Full-time, honours student).

To meet all assessment expectation for the practical components of the honours and master’s programme, some of the participants stated that the areas where they were placed did not provide adequate opportunities. These students had to consequently search for clinical learning opportunities outside of their clinical placements facilities, as one full-time master’s student participant explained: ‘There are no groups and families [for assessment of group and family therapy skills] in anonymous institution.’

### Theme 3: Research

The honours, master’s and doctoral students who were engaged in research activities explained that they had challenges with regard to information literacy and supervision. One honours student mentioned a challenge related to information literacy. Despite honours students also doing a research component, they did not mention any supervisory challenges. Supervisory challenges were mentioned by one master’s student and all three participating doctoral students.

#### Information literacy

The challenges experienced with information literacy related to the participant’s inadequate computer literacy and ability to access articles and other media electronically.

The participants reported that, prior to their enrolment in the postgraduate programme, they were not sufficiently computer literate and did not know how to access electronic literature. A honours participant also did not know how to independently access literature such as subject journals electronically on or off campus. One of the participants indicated: ‘Another challenge is the use of [a] computer in library searching for reading materials’ (full-time, honours student).

#### Supervisory relationship

Participants felt that some of the supervisors lacked passion for supervision, were unapproachable and rigid, and had a negative attitude towards them. These frustrations were voiced by mostly doctoral students:

The feeling that one perceives during the supervision is that one is being done a favor or is a burden, in spite of an appointment that has been made with that supervisor, e.g. a supervisor reminds one of the other appointments that he/she has with the other students or other people whilst the discussion is going on, which reduces [the students’] focus, motivation and concentration. (Part-time, doctoral student)

Another student explained: ‘[The] supervisor often does not respond to the e-mailed work and when reminded he/she sometimes shows less regard …’ (Part-time, doctoral student) and:

Lack of evidence of passion for supervision from [the] supervisor, hence as a student you feel as if you are a burden, with little or no external motivation from [the] supervisor. (Part-time, doctoral student)

#### Structure and process of supervision

Challenges in the supervision process and structure included late allocation of supervisors to students, lack of structure regarding supervision feedback as well as the perceived lengthy process for approval of research protocols. This was cited by some participants (mostly doctoral students) in the following quotations:

‘Not having a proper system for feedback and meetings.’ (Part-time, doctoral student 3)

‘Sometimes not receiving feedback in time.’ (Part-time, doctoral student 3)

‘Sometimes you leave the session confused because of a mammoth task to be addressed/attended to.’ (Part-time, doctoral student 1)

One master’s student commented on the long duration for the approval of research proposals.

To further highlight the challenges with regard to the guidance and sharing of information about the research process, some students made the following suggestions: ‘Clear guidelines from supervisor on how to submit the work for step-by step guidance and to facilitate acceptance by the relevant [ethics] committees…’ (part-time, doctoral student).

## Discussion

This study identified personal, institutional and research challenges as did a number of other studies (Clerehan et al. 2012; Essa 2011; Shen 2008).

Regarding personal challenges, the participants indicated about financial, employment, family and accommodation challenges. Perna (2004) explained that financial challenges are common and relate to the direct cost of education (e.g. university fees and accumulation of educational debt), the peripheral cost (cost of living away from home) and the inability to earn an income if the postgraduate student is not fully employed at the time. Mutula (2011:189) added that most countries on the African continent do not have funding for postgraduate research. Financial challenges contribute to the academic challenges as, with limited financial resources, students are unable to purchase data bundles to access literature through internet searchers (Roets & Maritz 2013). In the study by Cleary et al. (2011) the two main reasons for non-completion of doctoral degrees were financial and family stressors.

Postgraduate students in this study reported that travelling for long distances and lack of accommodation on campus were costly and time-consuming. They also mentioned their frustrations in finding accommodation within the institutional residences. Clerehan et al. (2012:220) concur with the participants’ challenges regarding accommodation by stating that early critical issues for students concerned finding accommodation and other settling-in processes.

The postgraduate students’ experiences of high workload at their place of employment, which affected their study performance, is in line with the challenges experienced by postgraduate students in other studies such as that reported by Essa (2011) who found that all the participants were working adults who also had other responsibilities, for example family (which included immediate and extended family members) and workplace commitments. Maasdorp and Holtzhausen (2011) support the challenge of workload experienced by the participants in this study by stating that students faced significant problems when beginning their postgraduate studies related to the worry about expectations, the break from study they have had and because of time in the workforce.

The participants in this study alluded to having experienced an imbalance regarding their studies, employment and family responsibilities. These experiences are supported by Essa (2011) who expounded that balancing of academic, work and family responsibilities is a challenge for postgraduate students who may be employed and have families of their own. Benshoff et al. (2015) added that fulfilling multiple roles, responsibilities and expectations is a common feature of postgraduate studies.

The educational experiences, mentioned by the participants in this study, comprise high workload with time constraints, inflexible contact sessions, inadequate subject information and limited time for interaction with other students. Barroca et al. (2010) explained that through effective induction, many challenges that arise from misconceptions or inappropriate expectations for these students might be bypassed. In support of the participants’ experiences of high workload with time constraints in this study, Essa (2011), Hunt, Mehta and Chan (2009), and Lessing and Schulze (2002) described that the limited time in the programme for student engagement and support contributes to a sense of isolation.

A participant in this study pointed out that they had challenges related to information literacy and supervision of research. They mentioned that they find it difficult to search for literature in the library, because they are not computer literate. However, they have realised that the use of the library is vital for them to pass their examinations and tests. Honey et al. (2006) emphasises that information literacy is required in postgraduate programmes and in nursing. However, many nurses who currently enrol in postgraduate studies are of an older generation that requires additional assistance in terms of information literacy. They should be able to use the newest research evidence in their daily practice and therefore developing postgraduate students’ information literacy as an essential part of postgraduate education. In the study by Essa (2011) it was also found that postgraduate students lack adequate computer literacy, typing skills and the ability to use the internet effectively.

The postgraduate students’ experiences of supervisors being unsupportive, unapproachable and rigid, and having a negative attitude towards them were expressed by the participants in this study. Similarly, Essa (2011) and Clerehan et al. (2012) found that many postgraduate students felt that the lecturers were distant, inaccessible, unapproachable and abrupt and this seemed to be the case whether the students stayed in the same or a different province than where the institution was situated. Frischer and Larsson (2000) and Wadesango and Machingambi (2011) concur with the importance of support by explaining that the supervisor–supervisee relationship is central to progress and completion.

When comparing the part-time and full-time student challenges in this study, as could be expected, employment challenges were more common for part-time students. In the research category there were more challenges mentioned by part-time students, but that was related to the fact that all three doctoral students were part-time students. Symons (2001) proposes that these challenges are worse for postgraduate coursework students, mature-aged students, international students and part-time students.

## Limitations

A limitation of the study was that only students who had email access were able to participate. Also, electronic responses limited the exploration of comments made by students in more detail through probing by the researchers. A future direction would be to include a much larger number of students from more than one university and doing in-depth individual interviews and focus groups. Also, more specific questions could be asked to explore themes identified in these data as well as themes that have emerged from the literature, but were not mentioned in these data (e.g. questions about language proficiency and deeper exploration of challenges related to information literacy).

## Recommendation

The financial needs of postgraduate students should be considered by universities providing adequate financial support and adequate access to information about available bursary and loan opportunities. Prior to students commencing, employers should be offered the schedule comprising the compulsory planned block dates to enable students to negotiate their study leave and days off with their employers.

Academic programmes should be reviewed on a regular basis and their relevance should be assessed through advisory committees. Credit values allocated to the programme and requirements by the South African Nursing Council should guide the workload. Workshops that empower students with learning skills and time management should be offered.

To ensure that information is distributed and thus students’ expectations of programmes are clear, orientation programmes (Polson 2003 & Rice 2003 as cited in Benshoff et al. 2015) should be implemented and effective platforms where information can be shared should be made available. Flexible teaching and delivery methods should be implemented, including satellite campuses, after-hours and weekend contact sessions, and the use of interactive e-learning platforms (Honey et al. 2006).

Peer group support should be mobilised by creating student support communities with strategies such as the use of dissertation partners, peer support groups, workshops and collaborative study groups (Benshoff et al. 2015; Conrad 2003; Juniper & Cooper 2002; Lategan 2008; Lessing & Schulze 2002; Topping et al. 2000).

## Conclusion

Personal, research as well as academic and institutional challenges were identified. These challenges are similar to studies conducted nationally and internationally such as the studies by Clerehan et al. (2012), Shen (2008) and Essa (2011). Despite no real differences were noticed between part-time and full-time students in this study (except on employment challenges), various programmes challenges should be explored by academic staff and support should be provided. To address these challenges and in so doing to improve completion, a culture of support for the postgraduate students should be established and maintained.

## References

[CIT0001] American Association of Colleges of Nursing, 2006, *The essentials of doctoral education for advanced nursing practice*, viewed 12 May 2015, from http://www.aacn.nche.edu/publications/position/DNPEssentials.pdf

[CIT0002] Anonymous, 2011, *Annual research report 2011, division of research development and administration*, Anonymous University, South Africa.

[CIT0003] Barroca L., Rapanotti L., Petre M., Vargas-Vera M. & Reeves A., 2010, *Developing research degrees online*, viewed 12 May 2015, from http://oro.open.ac.uk/25295/1/ICALT10submission.pdf

[CIT0004] Benshoff J.M., Cashwell C.S. & Rowell P.C., 2015, ‘Graduate students on campus: Needs and implications for college counselors’, *Journal of College Counselling* 18, 83–94. 10.1002/j.2161-1882.2015.00070

[CIT0005] Botma Y., Greeff M., Mulaudzi F.M. & Wright S.C.D., 2010, *Research in health sciences*, Heinemann, Cape Town.

[CIT0006] Cambridge University Press, 2017a, *The Oxford Dictionary*, viewed 10 March 2018, from http://dictionary.cambridge.org/dictionary/english/challenge

[CIT0007] Cambridge University Press, 2017b, *The Oxford Dictionary*, viewed 10 March 2018, from http://dictionary.cambridge.org/dictionary/english/postgraduate

[CIT0008] Cambridge University Press, 2017c, *The Oxford Dictionary*, viewed 10 March 2018, from http://dictionary.cambridge.org/dictionary/english/university?q=University

[CIT0009] Choo K.L., 2007, ‘The implications of introducing critical management education to Chinese students studying in UK business schools: Some empirical evidence’, *Journal of Further and Higher Education* 31(2), 145–158. 10.1080/03098770701267614

[CIT0010] Cleary M.E., Hunt G. & Jackson D., 2011, ‘Demystifying PhDs: A review of doctorate programs designed to fulfil the needs of the next generation of nursing professionals’, *Contemporary Nurse* 39(2), 273–280. 10.5172/conu.2011.39.2.273 22551438

[CIT0011] Clerehan R., McCall L., McKenna L. & Alshahrani K., 2012, ‘Saudi Arabian nurses’ experiences of studying Masters degrees in Australia’, *International Nursing Review* 59(2), 215–221. 10.1111/j.1466-7657.2011.00951.x 22591093

[CIT0012] Conrad L., 2003, *Five ways of enhancing the postgraduate community: Student perceptions of effective supervision and support*, Griffith University, Brisbane, viewed 12 May 2015, from http://www.herdsa.org.au/wpcontent/uploads/conference/2003/PDF/HERDSA57.pdf

[CIT0013] Council on Higher Education, 2013, *Higher education qualification sub-framework*, Council of Higher Education, Pretoria.

[CIT0014] Department of Education, 2001, *National plan for higher education*, Department of Education, Pretoria.

[CIT0015] Department of Higher Education and Training, 2015, *Research outputs policy*, viewed 16 June 2017, from http://www.dhet.gov.za/Policy%20and%20Development%20Support/Research%20Outputs%20policy%20gazette%202015.pdf

[CIT0016] De Vos A.S., Strydom H., Fouché C.B. & Delport C.S.L., 2011, *Research at grass roots: For the social sciences and human service professions*, 4th edn., Van Schaik, Pretoria.

[CIT0017] Essa I., 2011, ‘Reflecting on some of the challenges facing postgraduate nursing education in South Africa’, *Nurse Education Today* 31, 253–258. 10.1016/j.nedt.2010.11.007 21126811

[CIT0018] Frischer J. & Larsson K., 2000, ‘Laissez-faire in research education – An inquiry into a Swedish doctoral program’, *Higher Education Policy* 13(2), 132–155. 10.1016/S0952-8733(99)00022-7

[CIT0019] Haksever A.M. & Manisali E., 2000, ‘Assessing supervision requirements of PhD students: The case of construction management and engineering in the UK’, *European Journal of Engineering Education* 25(1), 19–32. 10.1080/030437900308616

[CIT0020] Holloway I. & Wheeler S., 2010, *Qualitative research in nursing and healthcare*, 3rd edn., Wiley, Oxford.

[CIT0021] Honey M., North N. & Gunn C., 2006, ‘Improving library services for graduate nurse students in New Zealand’, *Health Information and Libraries Journal* 23(2), 102–109. 10.1111/j.1471-1842.2006.00639.x 16706865

[CIT0022] Hunt M.R., Mehta A. & Chan L.S., 2009, ‘Learning to think qualitatively: Experiences of graduate students conducting qualitative health research’, *International Journal of Qualitative Methods* 8(2), 129–135. 10.1177/160940690900800204

[CIT0023] Juniper S. & Cooper G., 2002, ‘A workshop approach to postgraduate research training in generic skills and strategies’, paper presented at the Quality in Postgraduate Research: Integrating Perspectives Conference, Adelaide, 18–19 April 2002.

[CIT0024] Lategan L.O.K., 2008, ‘Theme 1: Why the fuss about research and postgraduate research?’, in Lategan L.O.K. (ed.), *An introduction to postgraduate supervision*, pp. 1–4, African Sun Media, Stellenbosch.

[CIT0025] Lessing A.C. & Schulze S., 2002, ‘Postgraduate supervision and academic support: Students’ perceptions’, *South African Journal of Higher Education* 16(2), 139–149. 10.4314/sajhe.v16i2.25253

[CIT0026] Lincoln Y.S. & Guba E.G., 1985, *Naturalistic inquiry*, Sage, Beverley Hills, CA.

[CIT0027] Maasdorp C. & Holtzhausen S.M., 2011, ‘Bridging the gap towards post graduate studies at the Central University of Technology, Free State’, *Interim: Interdisciplinary Journal* 10(1), 38–51.

[CIT0028] Mutula S.M., 2011, ‘Challenges of postgraduate research: Case of developing countries’, *South African Journal of Libraries and Information Science* 77(2), 184–190. 10.7553/77-2-60

[CIT0029] Polit C.T. & Beck D.F., 2012, *Nursing research: Generating and assessing evidence for nursing practice*, 9th edn., Lippincott Williams & Wilkins, Philadelphia, PA.

[CIT0030] Perna L.W., 2004, ‘Understanding the decision to enroll in graduate school: Sex and racial/ethnic group differences’, *The Journal of Higher Education* 75(5), 487–527. 10.1080/00221546.2004.11772335

[CIT0031] Public Health and Social Development Sectorial Bargaining Council, 2007, *Agreement on implementation of an occupational specific dispensation (OSD) for nurses: Resolution 3 of 2007*, Public Health and Social Development Sectoral Bargaining Council, Pretoria.

[CIT0032] Roets L. & Maritz J.E., 2013, ‘Challenges, opportunities and achievements of nurses’ research supervision across language borders’, *African Journal for Physical Health Education, Recreation and Dance*, suppl. ser. 2, 68–79.

[CIT0033] Rosseter R., 2012, *Nursing faculty shortage*, viewed 11 November 2012, from www.aacn.nche.edu/media-relations/fact/nursing-faculty-shortage

[CIT0034] Shen C., 2008, ‘Being and doing’ in a new academic environment: Challenges faced by seven Chinese post-graduate students at a South African University’, unpublished Masters research report, University of the Witwatersrand, Johannesburg.

[CIT0035] Symons M., 2001, *Starting a coursework postgraduate degree: The neglected transition*, viewed 01 January 2015, from learning.uow.edu.au/LAS2001/unrefereed/symons.pdf

[CIT0036] Topping K.J., Smith E.F., Swanson I. & Elliot A., 2000, ‘Formative peer assessment of academic writing between postgraduate students’, *Assessment and Evaluation in Higher Education* 25(2), 149–169. 10.1080/713611428

[CIT0037] Wadesango N. & Machingambi S., 2011, ‘Post graduate students’ experiences with research supervisors’, *Journal of Sociology and Social Anthropology* 2(1), 31–37. 10.1080/09766634.2011.11885545

